# Simultaneous monitoring of activity and heart rate variability in depressed patients: A pilot study using a wearable monitor for 3 consecutive days

**DOI:** 10.1002/npr2.12285

**Published:** 2022-07-29

**Authors:** Norie Koga, Yoko Komatsu, Ryo Shinozaki, Ikki Ishida, Yusuke Shimizu, Sonoko Ishimaru, Hiroshi Kunugi

**Affiliations:** ^1^ Department of Mental Disorder Research, National Institute of Neuroscience National Center of Neurology and Psychiatry Tokyo Japan; ^2^ Comfort Engineering Laboratory TOYOBO co., Ltd. Shiga Japan; ^3^ Development Department Union Tool, co. Niigata Japan; ^4^ Department of Psychiatry Teikyo University School of Medicine Tokyo Japan

**Keywords:** activity, heart rate variability, major depressive episode circadian rhythm, sex difference, wearable monitor

## Abstract

**Introduction:**

Reduced activity and sleep–wake rhythm disturbances are essential features of depressive episodes. In addition, alterations in heart rate variability (HRV) have been implicated in depression. By using a wearable sensor that monitors 3‐dimensional acceleration and HRV simultaneously, we examined the activity and HRV indices in depressive episode of mood disorders.

**Methods:**

Participants were 19 patients (13 major depressive disorder [MDD] and 6 bipolar depression; 11 females) and 18 controls (9 females) matched for age and ethnicity (all Japanese) who completed 3 consecutive days of all‐day monitoring by a small and light device attached to the chest.

**Results:**

Activity magnitude was significantly reduced while lying/resting time was increased in depressed patients, compared with controls. When males and females were examined separately, male, but not female, patients showed significant reduction in activity. HRV indices such as R‐R interval and high‐frequency power (a parameter for the parasympathetic system) were significantly decreased in patients than in controls. Significant differences in activity and HRV indices were seen only in males. Sympathetic load during sleep significantly correlated with damped rest–activity rhythm in depressed patients.

**Limitations:**

The number of participants was small, and the majority of the participants were taking psychotropic medications.

**Conclusions:**

We obtained evidence for reduced activity, increased lying/resting time, and reduced HRV indices in male depressed patients. The simultaneous monitoring for activity and HRV suggested greater sympathetic load during sleep is associated with damped rest–activity rhythm (increased activity during sleep and decreased daytime activity), which might be a characteristic pathology of depression.

## Abstract 2

By using a wearable sensor we examined 3‐dimensional acceleration and heart rate variability (HRV) simultaneously in depressive episode of mood disorders. We found reduced activity and altered HRV indices in depressed patients, compared with healthy participants, particularly in males. Moreover, we found that sympathetic load during sleep significantly correlated with damped rest‐activity rhythm in depressed patients, which might be a characteristic pathology of the illness.

## Graphical abstract

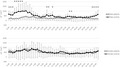

**Abbreviations**
ACT: average activity per day
ANOVA: analysis of variance
BD: bipolar disorder
CPeq: chlorpromazine‐equivalent dose
CVRR: coefficient of variation of the R‐R interval
DIAeq: diazepam‐equivalent dose
HAMD‐17: 17‐item version of the Hamilton Depression Rating Scale
HF: high frequency
HRV: heart rate variability
IMIeq: imipramine‐equivalent dose
L5: average activity across the least active 5‐h period of 24‐h sleep–wake rhythm
LF: low frequency
LT: lying time
M10: average activity during the most active 10‐h period
MDD: major depressive disorder
MINI: Mini International Neuropsychiatric Interview
NCNP: National Center of Neurology and Psychiatry
NLT: average number of lying time per day
NN50: numbers of successive RR intervals that differ by more than 50 ms
NRT: average number of resting time per day
pNN50: percentage of differences between RR intervals with an absolute value greater than 50 ms
RA: relative amplitude of the rest–activity rhythm
RMSSD: square root of the mean of squared differences between successive beat intervals
RRI: RR interval
RT: resting time
SDNN: standard deviation of RR intervals
TLT: average total lying time per day
TRT: average total resting time per day
ULF: ultra‐low frequency
VLF: very low frequency

## 1 INTRODUCTION

Disturbances in circadian rest–activity rhythms are key features of depressed episode in patients with mood disorders.^1^, ^2^ Such disturbances can be objectively monitored by an actigraph (accelerometer) which has been used by many previous studies and revealed reduced activity and sleep disturbances in patients with mood disorders (reviewed by Tazawa et al.^3^). Previously, we also examined 24‐h activity rhythm by using an actigraph and reported reduced activity and poor sleep quality in depressed patients.^4^

Heart rate variability (HRV), another physiological measure that can be monitored by a wearable sensor on subtle changes in duration of successive heartbeats, that is, the interval between successive R waves of electrocardiogram (called RR interval: RRI), has been known to provide clinically useful information on autonomic nervous system functions, that is, those of the sympathetic and parasympathetic systems.^5^ Previous studies have shown reduced HRV in patients with major depressive disorder (MDD) and those with bipolar disorder (BD).^6^, ^7^, ^8^, ^9^, ^10^ Of note, a large meta‐analysis on HRV studies in healthy participants revealed that females had a significantly lower mean RRI and standard deviation of RRIs (SDNN).^11^ The power spectral density of HRV in females is characterized by significantly less total power that contains significantly greater high‐ (HF) and less low‐frequency (LF) power. Further, a recent study suggested that the effect of clinical depression on HRV measures is also sex‐dependent (eg, Kuang et al.^12^). These findings indicate the importance of sex differences when studying HRV in mood disorders.

Recent advances in wearable sensor technology have made it possible to monitor activity and HRV simultaneously. To our knowledge, however, no studies have thus far examined these objective physiological measures simultaneously in depression. Such simultaneous monitoring would be advantageous because it can obtain two physiological markers (activity and HRV), and it enables us to examine the possible relationship between the two. The present study aimed to examine whether 24‐h activity and HRV are altered in patients with a major depressed episode, compared with healthy controls. We tried to identify the time and magnitude of the differences between the patients and controls by monitoring 3 consecutive days. Based on the literature, we also paid attention to the issue of sex differences.

## 2 METHODS

### 2.1 Participants

Participants were recruited at the National Center of Neurology and Psychiatry (NCNP), through advertisements in a local free magazine or the announcements on our website. The participants were screened to have any major psychiatric disorders using the Japanese 5.0.0 version of Mini International Neuropsychiatric Interview (MINI)^13^, ^14^ by a trained research psychologist. Diagnosis was determined according to the Diagnostic and Statistical Manual of Mental Disorders 5th edition criteria (DSM‐5),^15^ based on information obtained through the MINI, additional interviews and medical charts if available. Only outpatients who met the DSM‐5 criteria for a current major depressive episode and were scored 15 or more by the GRID‐Hamilton Depression Rating Scale 17‐item version (HAMD‐17)^16^, ^17^ were enrolled in the study. In this pilot study, we included not only patients with MDD but also those with BD since we observed similar alterations in 24‐h activity rhythms for MDD and BD patients.^4^ Healthy controls had no current psychiatric illness according to the MINI and had no history of contact with any psychiatric services. Those individuals who had a medical history of neurological diseases, severe head injury, substance abuse, mental retardation, or severe medical illness were not enrolled in the study. Every participant gave written informed consent after receiving the study explanation. The study protocol was approved by the ethics committee at the NCNP and was carried out according to the Declaration of Helsinki.^18^

Initially, we recruited 22 patients with major depressive episode and 20 controls. However, we excluded 5 participants from the analyses because one patient (ID 24) did not wear the monitor at all, 2 patients (ID 8 and 41) and one control (ID 36) stopped wearing before 72 h, and one control (ID 5) presented fever during the experimental period. The remaining 19 patients (13 MDD and 6 BD) and 18 controls matched for age, sex, and ethnicity (all Japanese) completed the 72‐h monitoring.

### 2.2 Clinical and psychological assessments

Of the 19 patients, 13 were diagnosed as MDD and 6 as BD (all currently in a major depressive episode). For all patients with BD, their manic symptoms were in remission as defined by the total score on the Young Mania Rating Scale^19^ of 12 or less (range: 0–6; median: 2). Daily doses of antipsychotics, antidepressants, and benzodiazepines were converted to the chlorpromazine‐equivalent (CPeq), imipramine‐equivalent (IMIeq), and diazepam‐equivalent (DIAeq) doses, respectively, by the Japanese guideline.^20^

### 2.3 Heart rate variability and activity data acquisition and processing

#### 2.3.1 Sensor

Participants were asked to complete 3 consecutive days of all‐day monitoring under free‐living conditions by a small and light wearable HR sensor (40.8 × 37.0 × 8.9 mm; weight: 13 gram), myBeat WHS‐1 (Union Tool, co., Tokyo, Japan), which could also monitor 3‐dimensional acceleration. Although some actigraph studies on activity recommended recordings of 5 days or more (eg, Luik et al.^21^ and Goncalves et al.^22^), we chose 3 days' monitoring because our device was chest‐worn, but not wrist‐worn, which was substantially burdensome for the participants. Moreover, the battery of our device lasts no longer than 5 days. Initially, the examiner determined the best place on the subject's chest in order to record the RRI. Two disposable gel electrodes, BlueSensor (Ambu, Ballerup, Copenhagen, Denmark), were attached to the appropriate position and the monitor was bound to the electrodes. The RRI was measured by using the peak position of R wave with a resolution of 1 ms. The participants were instructed to wear it from 15:00 on the first experimental day to 15:00 on the 4th day except when they were showering or bathing. Data for such non‐wear period were treated as missing data. When we averaged the activity data, for example, we calculated mean activity level only when the participants were wearing the device. Alcohol intake was prohibited during the measurement. The 3‐axis acceleration was measured by MEMS accelerometer with 32 Hz and 8 bits in a dynamic range of ±4 G, where the unit “G” is the gravitational acceleration. WHS‐1 recorded a maximum acceleration every 4 s.

The activity magnitude was estimated by the equation below:

$$\text{Activity}=\sqrt{\left(x^{2}+y^{2}+z^{2}\right)}-1$$

where x, y, and z were acceleration of X (left–right), Y‐axis (vertical) and Z (anterior–posterior) axes. We considered that the participant was lying (ie, in a supine position) when y > −0.7G (see the supplementary methods) according to previous studies in Japanese.^23^, ^24^ Then we defined a priori “lying time (LT)” when y > −0.7G lasted for 10 min or more and “resting time (RT)” when y > −0.7G lasted for 1 h or more. We calculated mean activity per day (ACT), mean total LT per day (TLT), and average number of LT per day (NLT), mean total RT per day (TRT), and average number of RT per day (NRT) for each subject. Further, we calculated L5 (defined as the average activity across the least active 5‐h period of 24‐h sleep–wake rhythm; higher values indicate restlessness), M10 (the average activity during the most active 10‐h period; low levels indicating inactivity during daytime), and RA (relative amplitude of the rest–activity rhythm that was calculated as the difference between M10 and L5 divided by M10 plus L5; RA standardizes for activity‐level differences across subjects and reflects strength of circadian signal; values closer to 1 represent rhythms with higher relative amplitudes).

#### 2.3.2 Heart rate variability data

RRI data were obtained and subject to fast Fourier transform by using the RRI analyzer software (Union Tool, co., Tokyo, Japan), which generated power spectra from which we obtained ultra‐low frequency (ULF: 0.0033 Hz or less), very low frequency (VLF: 0.0033 to 0.04 Hz), LF (0.04 to 0.14 Hz), and HF (0.14 to 0.4 Hz) bands. We also calculated average CVRR (coefficient of variation of the RRI), SDNN (standard deviation of normal to normal [NN] inter‐beat interval [IBI]) and RMSSD (square root of the mean of squared differences between successive beat intervals), NN50 (numbers of successive RR intervals that differ by more than 50 ms), pNN50 (percentage of differences between RR intervals with an absolute value greater than 50 ms). According to the literature,^25^, ^26^ the indices of HRV could be summarized as follows. LF is influenced by both sympathetic and parasympathetic activities, while HF is affected by mostly parasympathetic activities. LF/HF ratio implicates the sympathetic predominance compared with parasympathetic activities. SDNN is considered to reflect both sympathetic and parasympathetic functioning, whereas CVRR, RMSSD, and pNN50 have relevance to parasympathetic functioning. Methods of the wearable monitoring are described in more detail in supplementary methods.

### 2.4 Statistical analyses

Data collected over the 3‐day period were averaged into a single 24‐h profile for analyses. Categorical variables were compared by the chi‐square test, while differences in continuous demographic and clinical variables were compared by Student's or Welch's *t*‐test between two independent groups. To be conservative, comparisons in activity and HRV measures between patients and controls were conducted by the non‐parametric Mann–Whitney U test since some of activity and HRV data did not show normal distribution. Since sex differences were reported in HRV indices,^11^ we compared activity and HRV measures for each sex as well. We have made additional analyses by 2‐way (sex and diagnosis) analysis of variance (ANOVA) on variables which were not deviated from the normal distribution based on the Shapiro–Wilk test, controlling for age. The possible correlation between activity and HRV indices was examined by Spearman's correlational analysis. The effect sizes were evaluated by *Cramer*'*s V* for the chi‐square test, *Cohen*'*s d* for the unpaired two‐sample *t*‐test, *η* ^*2*^ for Mann–Whitney U test. The statistics were calculated by using the Statistical Package for the Social Sciences version 26.0 (SPSS Japan, Tokyo, Japan). All the statistical tests were two‐tailed, and *P‐*values of less than 0.05 were deemed significant.

## 3 RESULTS

**FIGURE 1:**
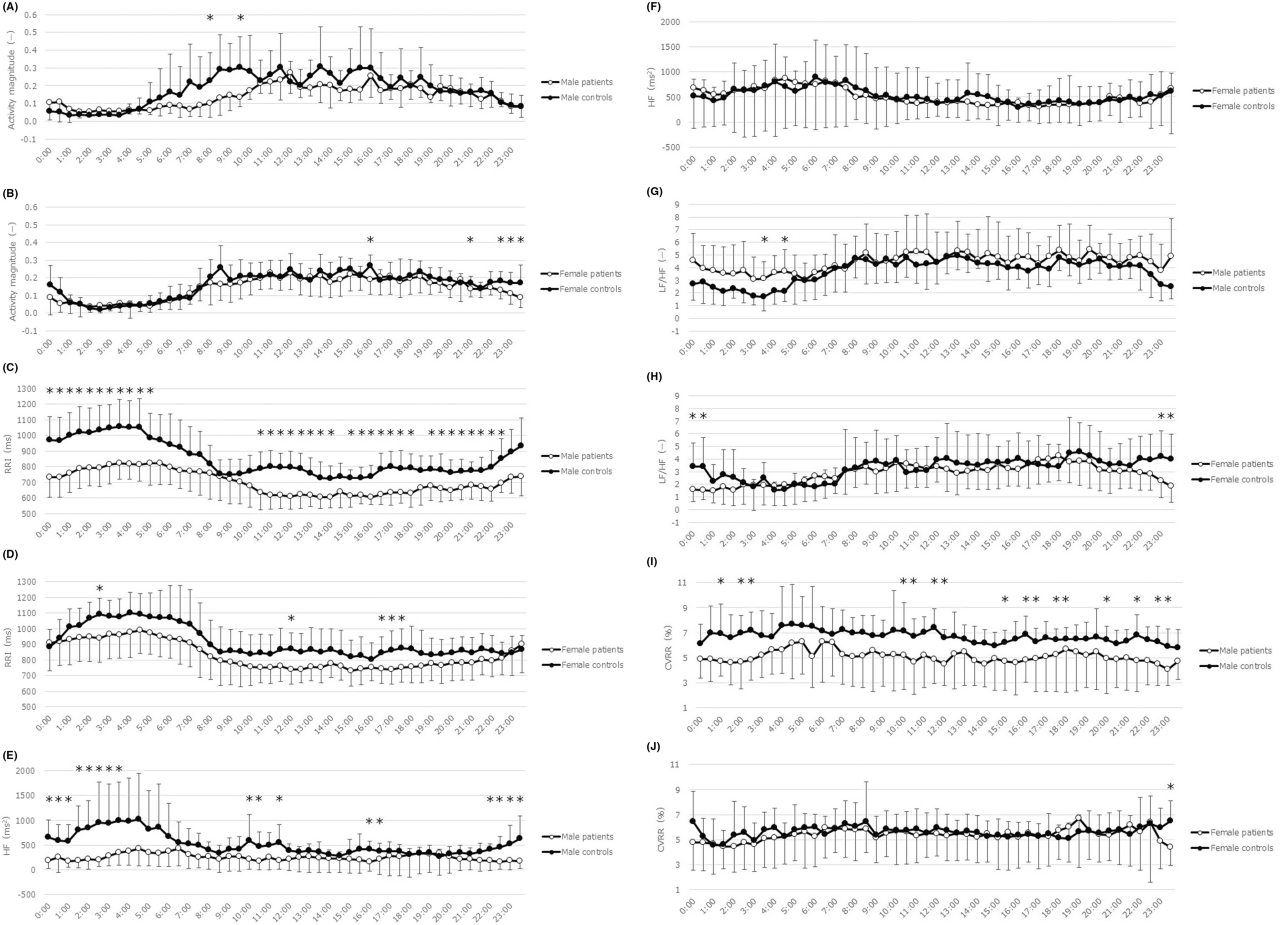
Diurnal variations of activity and heart rate variability indices in depressed patients and controls stratified by sex. Activity magnitude male (A) and female (B); RRI: R‐R interval male (C) and female (D); HF: high‐frequency bands male (E) and female (F); LF/HF: ratio of low frequency (LF) bands to HF bands male (G) and female (H); CVRR: coefficient of variation of the RRI male (I) and female (J). Average of 3 consecutive days. *: uncorrected *P* < 0.05 by Mann–Whitney U test

Demographic and clinical characteristics of the participants included in the analysis are shown in Table 1. There was no significant difference in the distributions of age, sex, body mass index, or the rate of smoker, between the patients and controls, although the rate of smoker tended to be higher in the patients than in the controls. The majority of patients (n = 16, 84%) were using psychotropic medications. Figure 1 shows diurnal variations of activity and HRV indices in depressed patients and controls stratified by sex.

**TABLE 1:** Demographic and clinical characteristics of the participants

	Depressed patients (*n* = 19)		Healthy controls (*n* = 18)		Statistical comparison
	Mean ± SD	Range	Mean ± SD	Range	
Age (years)	42.3 ± 12.5	27–64	43.5 ± 8.4	29–57	*Welch*'*s t*(580.8) = 1.10, *P* = 0.27, *Cohen*'*s d* = 0.09
Sex, male (%)	7 (36.8)		9 (50.0)		*χ* ^*2*^(1) = 0.28, *p* = 0.87, *Cramer*'*s V* = 0.01
Education (years)	14.8 ± 2.7	9–20	15.9 ± 2.9	12–23	*Student*'*s t*(599) = −0.30, *P* = 0.76, *Cohen*'*s d* = 0.02
Body mass index (kg/m^2^)	22.4 ± 4.3	16–30	22.3 ± 3.5	18–32	*Welch*'*s t*(580.8) = 1.39, *P* = 0.17, *Cohen*'*s d* = 0.11
Smoking (%)	6 (31.6)		1 (5.6)		*P* = 0.09, Fisher's exact test, *Cramer*'*s V* = 0.33
Age of onset (years)	32.6 ± 12.0	14–60			
Duration of illness (years)	6.2 ± 5.9	0–22			
Psychotropic medication use (%)	16 (84.2)				
Antipsychotics use (%)	11 (57.9)				
CPeq total (mg/day, *n* = 11)	86.0 ± 119.0	25–403			
Antidepressant use (%)	11 (57.9)				
IMIeq (mg/day, *n =* 11)	129.9 ± 151.6	50–450			
Minor tranquilizer use (%)	10 (52.6)				
DIAeq (mg/day, *n =* 10)	3.6 ± 4.8	3–18			
HAMD‐17	22.0 ± 4.1	16–31			

CPeq, chlorpromazine‐equivalent dose; DIAeq, diazepam‐equivalent dose; HAMD‐17,17‐item version of the Hamilton Depression Rating Scale; IMIeq, imipramine‐equivalent dose; SD, standard deviation.

### 3.1 Activity

First, we examined the possible relationships of activity variables with age and sex. There was no significant correlation of age with any activity data in the patients (data not shown). However, there was a significantly negative correlation or a trend for a negative correlation with TLT (Spearman's ρ = −0.69, *P* = 0.002), NLT (ρ = −0.45, *P* = 0.063), TRT (ρ = −0.68, *P* = 0.002), and NRT (ρ = −0.52, *P* = 0.026) in the controls, although there was no significant correlation with ACT (ρ = 0.12, *P* = 0.64), L5 (ρ = −0.05, *P* = 0.85), M10 (ρ = 0.08, *P* = 0.74), and RA (ρ = 0.05, *P* = 0.85). The observed negative correlations were in line with the well‐known phenomenon that sleep time gets shorter along with aging in healthy people of the current age range (eg, Ohayon et al.^27^). There were no significant differences in ACT between males and females in the patients (U = 37, *P* = 0.71) or in the controls (U = 30, *P* = 0.39). No significant sex differences were found for any other activity variable (ie, TLT, NLT, TRT, NRT, L5, M10, or RA) (data not shown).

Table 2 shows results of comparisons in activity indices between the patients and controls. In all participants, there were significant differences in ACT, TLT, NLT, and NRT between the two groups. There was a similar trend in TRT. These results indicate that activity magnitude was decreased, while time and number of lying and resting periods were increased in depressed patients. When males and females were compared individually, significant decrease was found only for NLT, NRT, and RA in males. There was no significant difference in any activity index in females.

**TABLE 2:** Comparisons in activity indices between depressed patients and controls

	Patients (7 M, 12 F)		Controls (9 M, 9 F)		Comparison (Mann–Whitney)		
	Median	Interquartile range	Median	Interquartile range	*U*	*P*	η^2^
1. ACT							
Total	0.14	(0.13–0.15)	0.165	(0.13–0.19)	99.5	**0.029**	0.133
Male	0.14	(0.13–0.14)	0.19	(0.13–0.22)	17.0	0.142	0.161
Female	0.14	(0.11–0.17)	0.15	(0.135–0.185)	38.0	0.277	0.066
2. TLT (min)							
Total	605.0	(486.7–710.3)	476.2	(389.0–572.9)	244.0	**0.026**	0.137
Male	617.7	(552.0–682.7)	515.0	(401.2–594.8)	49.0	0.071	0.229
Female	534.8	(477.9–753.6)	471.0	(369.8–583.2)	78.0	0.095	0.146
3. NLT							
Total	3.67	(2.33–5.33)	2.00	(1.00–2.67)	267.5	**0.003**	0.241
Male	5.0	(3.33–5.67)	1.67	(1.00–2.33)	56.5	**0.005**	0.472
Female	3.33	(2.33–4.00)	2.67	(1.33–3.17)	72.0	0.219	0.083
4. TRT (min)							
Total	514.0	(472.0–677.3)	453.5	(378.2–559.4)	228.0	0.086	0.083
Male	559.0	(472.0–670.3)	515.0	(384.0–580.0)	40.0	0.408	0.054
Female	490.0	(446.8–707.2)	448.7	(353.3–543.7)	75.0	0.148	0.111
5. NRT							
Total	1.67	(1.00–3.00)	1.00	(1.00–1.75)	224.0	**0.026**	0.147
Male	2.00	(1.33–2.67)	1.00	(1.0–1.5)	51.0	**0.042**	0.312
Female	1.50	(1.00–3.00)	1.33	(1.00–2.00)	69.0	0.310	0.061
6. L5							
Total	0.042	(0.023–0.051)	0.033	(0.023–0.048)	183.0	0.715	0.004
Male	0.049	(0.043–0.053)	0.033	(0.019–0.056)	44.5	0.174	0.118
Female	0.029	(0.017–0.049)	0.033	(0.026–0.044)	42.0	0.422	0.035
7. M10							
Total	0.211	(0.183–0.235)	0.237	(0.197–0.285)	117.5	0.105	0.071
Male	0.211	(0.186–0.217)	0,0.258	(0.182–0.378)	19.0	0.210	0.109
Female	0.219	(0.144–0.255)	0.210	(0.196–0.278)	42.0	0.422	0.035
8. RA							
Total	0.65	(0.59–0.80)	0.74	(0.65–0.83)	124.0	0.159	0.055
Male	0.61	(0.56–0.67)	0.76	(0.67–0.87)	11.0	**0.031**	0.294
Female	0.75	(0.63–0.86)	0.73	(0.64–0.81)	58.0	0.808	0.004

ACT: mean activity per day; L5: average activity across the least active 5‐h period; M10: average activity during the most active 10‐h period; NLT: average number of lying time per day; NRT: average number of RT per day; RA: relative amplitude of the rest–activity rhythm; TLT: mean total Lying time per day; TRT: mean total resting time per day. Significant *P* values are described in bold cases.

In addition, we performed 2‐way (sex and diagnosis) ANOVA analyses on ACT, TRT, L5, M10, and RA controlling for age, since data of these variables in healthy participants were not deviated from the normal distribution (*P* > 0.05 by the Shapiro–Wilk test). We found significant effects of diagnosis on ACT and M10 (Table S1), which provides support for decrease in overall and daytime activity in depressed patients.

Figure 1A shows comparisons of diurnal variation of activity for males and females separately, which illustrates the sex difference. In males, there was a marked difference in activity in the morning between the patients and controls, while an attenuated difference in activity was seen in the prebedtime in females. Notably, activity between 1 a.m. and 4 a.m. was increased rather than decreased, though statistically nonsignificant, in the patients than in the controls, indicating poorer sleep quality in the former.

Then, we examined the possibility that the observed decrease in activity of the patients might be related to medication. There was no significant correlation of IMIeq, DIAeq, or CPeq with any of the activity indices (all *P* > 0.05), although a non‐significant positive correlation was observed between IMIeq and NRT (Spearman's ρ = 0.41, *P* = 0.079).

### 3.2 Heart rate variability

There was no significant correlation of age with any HRV index (data not shown) in the patients, although a negative correlation at a trend level was observed for CVRR (Spearman's ρ = −0.417, *P* = 0.075) and SDNN (ρ = −0.395, *P* = 0.096). In contrast, there was a significantly negative correlation or its trend with HF (ρ = −0.414, *P* = 0.088), CVRR (ρ = −0.470, *P* = 0.049), SDNN (ρ = −0.490, *P* = 0.039), NN50 (ρ = −0.600, *P* = 0.008), and pNN50 (ρ = −0.522, *P* = 0.018) in controls, although such a correlation was not found for other indices (ie, RRI, LF/HF, VLF, ULF, or RMSSD) (data not shown). There were significant differences in RRI (U = 13.5, *P* = 0.013) and HF/LF (U = 14.0, *P* = 0.017) between males and females in the patients. However, there was no significant sex difference for any HRV index in the controls (data not shown).

Table 3 shows results of comparisons in HRV indices between the patients and controls. In the total participants, RRI, HF, and SDNN were significantly decreased in the patients than in the controls. There was a similar trend in ULF, RMSSD, and CVRR. Then, we made comparisons for each sex. In males, significant differences in the same directions were found for RRI, HF, and SDNN and a difference at a trend level was found for CVRR. In females, in contrast, differences did not reach statistical significance or trend level for any HRV index. Figure 1C–J shows comparisons of diurnal fluctuations of HRV indices (RRI, HF, LF/HF, and CVRR) for males and females, which illustrates the sex difference. In general, the differences between the patients and controls were greater in males than in females throughout the 24‐h day. In males, marked differences were observed during sleep time. In females, in contrast, the difference between the patients and controls were substantially attenuated. Notably, LF/HF values were decreased between 22 p.m. and 2 a.m. in female patients than in female controls, whereas those of male patients were increased than male controls during the same hours, which is another sex difference observed in our participants.

**TABLE 3:** Comparisons in heart rate variability indices between depressed patients and controls

	Patients (7 M, 12 F)		Controls (9 M, 9 F)		Comparison (Mann–Whitney)		
	Median	Interquartile range	Median	Interquartile range	*U*	*P*	η^2^
1. RRI							
Total	789.0	(669.0–900.0)	892.5	(827.0–929.3)	93.0	***P* = 0.017**	0.156
Male	669.0	(648.0–789.0)	885.0	(799.0–905.5)	6.0	***P* = 0.005**	0.203
Female	867.5	(764.5–904.8)	896.0	(839.0–1022.5)	34.0	*P* = 0.169	0.056
2. HF							
Total	356.0	(214.0–578.0)	570.0	(455.5–935.0)	99.0	***P* = 0.029**	0.133
Male	226.0	(71.0–578.0)	573.0	(469.5–864.5)	10.0	***P* = 0.023**	0.144
Female	377.5	(284.5–949.3)	567.0	(307.0–1035.0)	41.0	*P* = 0.382	0.023
3. LF/HF							
Total	4.0	(3.5–4.5)	4.0	(2.9–4.9)	166.5	*P* = 0.893	0.001
Male	4.5	(4.0–6.8)	4.3	(3.4–4.9)	40.5	*P* = 0.351	0.025
Female	3.7	(2.5–4.2)	3.8	(2.4–5.8)	52.0	*P* = 0.917	0.001
4. VLF							
Total	1299.0	(541.0–3072.0)	2248.0	(1269.3–3192.8)	122.0	*P* = 0.142	0.062
Male	919.0	(377.0–3111.0)	2487.0	(1209.5–3196.5)	21.0	*P* = 0.299	0.034
Female	1384.5	(589.8–2926.5)	2054.0	(1204.0–3345.5)	39.0	*P* = 0.310	0.032
5. ULF							
Total	6.0	(3.0–12.0)	9.0	(6.8–15.8)	115.0	*P* = 0.092	0.081
Male	4.0	(3.0–14.0)	10.0	(7.0–16.5)	22.0	*P* = 0.351	0.028
Female	7.0	(2.3–11.5)	9.0	(6.0–17.0)	38.0	*P* = 0.277	0.036
6.SDNN							
Total	35.1	(25.2–45.2)	50.7	(42.8–60.2)	99.0	***P* = 0.029**	0.133
Male	35.0	(19.0–44.5)	53.9	(40.8–63.2)	12.0	***P* = 0.042**	0.118
Female	37.8	(25.7–62.2)	48.5	(38.2–56.8)	37.0	*P* = 0.247	0.041
7. RMSSD							
Total	25.4	(18.3–35.8)	35.8	(27.8–46.4)	111.0	*P* = 0.070	0.092
Male	25.4	(9.2–35.8)	35.6	(26.2–40.6)	16.0	*P* = 0.114	0.075
Female	25.1	(21.7–51.6)	36.4	(25.3–55.5)	40.0	*P* = 0.345	0.047
8. CVRR							
Total	5.2	(3.4–5.8)	6.4	(5.6–7.2)	107.0	*P* = 0.053	0.053
Male	5.2	(2.9–5.7)	6.6	(5.7–7.6)	13.5	*P* = 0.055	0.101
Female	5.2	(3.4–7.4)	5.7	(4.8–7.2)	41.0	*P* = 0.382	0.024
9. NN50							
Total	3.7	(1.4–10.5)	7.6	(3.5–11.0)	124.5	*P* = 0.159	0.055
Male	4.5	(0.2–10.5)	7.7	(5.2–10.2)	20.0	*P* = 0.252	0.041
Female	3.4	(1.6–14.5)	6.1	(2.6–14.1)	45.0	*P* = 0.554	0.011
10. pNN50							
Total	6.0	(3.0–13.0)	12.5	(5.8–19.5)	126.0	*P* = 0.178	0.052
Male	7.0	(0.0–13.0)	13.0	(7.5–18.0)	18.5	*P* = 0.174	0.054
Female	5.5	(3.3–28.0)	11.0	(4.5–23.5)	46.0	*P* = 0.602	0.009

Significant *P* values are described in bold cases.

In addition, we performed 2‐way (sex and diagnosis) ANOVA analyses on RRI, HF, SDNN, RMSSD, CVRR, NN50, and pNN50, controlling for age. Since data of these variables in healthy participants were not deviated from the normal distribution (*P* > 0.05 by the Shapiro–Wilk test), We found significant effects of diagnosis on RRI, SDNN, CVRR, but not on HF, RMSSD, and NN50 (Table S2), which was similar results obtained by the non‐parametric analysis described above except only for HF (significant according to Mann–Whitney test, but not to ANOVA).

Furthermore, we compared HRV indices during L5 to control for activity. Results were essentially unchanged from those of the average data of 72 h except for LF/HF in males (significant increase in male patients than in male healthy participants in L5 [*P* = 0.005 by Mann–Whitney test], but not in average of 72 h [*P* = 0.351]), which accords with Figure 1G.

Then we examined the possible correlation between HRV indices and psychotropic medications in the patients. There was a significant correlation or its trend of IMIeq with RRI (Spearman's ρ = −0.58, *P* = 0.010), LF/HF (ρ = −0.48, *P* = 0.039), CVRR (ρ = −0.58, *P* = 0.009), SDNN (ρ = −0.61, *P* = 0.006), RMSSD (ρ = −0.55, *P* = 0.014), NN50 (ρ = −0.68, *P* = 0.002), and pNN50 (ρ = −0.62, *P* = 0.005). In line, there was a correlation at a trend level between IMIeq and HF (ρ = −0.45, *P* = 0.052). In contrast, there was no significant correlation of any HRV index with CPeq or DIAeq (all *P* > 0.1; data not shown).

### 3.3 Correlation analysis between activity and HRV

When correlations between activity and HRV indices were examined, there were no significant correlations for any combination of the two indices for average data of 72 h (all *P* > 0.05) (data not shown). However, to control for activity, we examined correlations of time‐dependent activity measures (L5, M10, and RA) with HRV indices during L5. We found significant correlations of L5 activity with RRI (ρ = −0.53, *P* = 0.001) and LF/HF (ρ = 0.44, *P* = 0.007) during L5 and those of a trend level with SDNN (ρ = −0.29, *P* = 0.078), RMSSD (ρ = −0.29, *P* = 0.086), NN50 (ρ = −0.27, *P* = 0.099), and pNN50 (ρ = −0.31, *P* = 0.058) in the total participants (Table S3). RA showed similar correlations with HRV indices during L5. When patients and controls were examined separately Table 4, we found significantly positive correlation between RRI and RA, that between LF/HF and L5, significantly negative correlations of LF/HF with M10 and RA in the patients. In contrast, we found a significantly negative correlation between RRI and L5 and a positive correlation between RRI and RA. No significant correlation of LF/HF with any time‐dependent activity measures was detected in the controls.

**TABLE 4:** Correlation of time‐dependent activity (L5, M10, and RA) with heart rate variability indices during L5 in patients and controls

Patients (*n* = 19)									
		RRI	HF	LF/HF	CVRR	SDNN	RMSSD	NN50	pNN50
Activity_L5	ρ	−0.420	−0.326	0.504	−0.114	−0.158	−0.287	−0.282	−0.294
	*P*	0.073	0.17	**0.028**	0.64	0.52	0.23	0.24	0.22
Activity_M10	ρ	0.312	0.323	−0.495	0.177	0.267	0.207	0.161	0.174
	*P*	0.19	0.18	**0.031**	0.47	0.27	0.40	0.51	0.48
Activity_RA	ρ	0.491	0.419	−0.64	0.216	0.270	0.340	0.312	0.332
	*P*	**0.033**	0.074	**0.0031**	0.37	0.26	0.15	0.19	0.17
Controls (*n* = 18)									
		RRI	HF	LF/HF	CVRR	SDNN	RMSSD	NN50	pNN50
Activity_L5	ρ	−0.683	−0.039	0.219	0.048	−0.400	−0.169	−0.157	−0.228
	*P*	**0.0018**	0.88	0.38	0.85	0.10	0.50	0.53	0.36
Activity_M10	ρ	0.230	0.096	0.321	0.197	0.135	0.077	0.044	0.069
	*P*	0.36	0.70	0.19	0.43	0.59	0.76	0.86	0.79
Activity_RA	ρ	0.717	0.082	0.026	0.051	0.410	0.199	0.164	0.232
	*P*	**0.0008**	0.75	0.92	0.84	0.091	0.43	0.52	0.35

*Note*: Sperman's correlation coefficients (ρ) and *P*‐values are shown.

Significant *P*‐values are described in bold cases.

Heart rate variability indices during L5 (least active 5 h for each participant) were examined.

Analyses on VLF and ULF are omitted.

## 4 DISCUSSION

We examined activity and HRV simultaneously by a light wearable monitor attached to the chest for 3 consecutive days. To our knowledge, this is the first study that examined diurnal courses of these physiological measures simultaneously in patients with major depressive episodes in comparison with healthy controls.

As expected, we found that activity indices were all lower in depressed patients than in controls. When each sex was examined, however, some indices (NLT and NRT) reached the statistical significance only in males while none of the indices did in females. The observation of reduced mean activity in patients compared with controls is as expected and in accordance with a number of previous studies (reviewed in Tazawa et al.^3^). In the present study we defined LT and RT based on the previous studies^23^, ^24^ that the participant was considered lying (ie, in a supine position) when y > −0.7. As a result, depressive patients showed increased number and total time for LT and RT, compared with controls. All the indices except for SRT reached the statistical significance in the total sample. The lowest p‐value was obtained for NLT (*P* = 0.003), indicating that NLT could be a good index to monitor inactivity in depressed patients particularly in males (*P* = 0.005).

When each sex was examined, the differences of any index did not reach the statistical significance in females in spite that the number of female participants was slightly greater than that of male participants. However, all the activity indices showed in the same direction, that is, more inactive in female patients than in female controls. It is therefore likely that the difference between patients and controls might be greater in males than in females and that the difference in females will also reach significance if the sample size was amplified. This is supported by the finding of no significant interaction between sex and diagnosis in ANOVA analyses on any activity indices. Although there is little information in the literature, one study reported that damped circadian amplitude of activity was observed only girls among depressed children and adolescents.^28^ When the diurnal variation of activity was examined (Figure 1A,B), male patients showed inactivity in the morning. On the other hand, female patients showed inactivity in prebedtime. Although such a difference between males and females are intriguing, further studies will be required to draw any conclusion.

Regarding HRV, we found significant differences in the HRV indices (RRI, HF, and SDNN) and differences at a trend level (ULF, RMSSD, and CVRR) in the total sample. These findings are generally in accordance with previous studies.^6^, ^7^, ^8^, ^9^, ^10^ Although the previous studies showed significant increase in LF/HF, we found no significant difference in LF/HF between patients and controls. Based on a recent meta‐analysis of Koch et al.,^10^ the effect size of LF/HF (Hedge's *g* = 0.195) was substantially lower than that of other indices such as HF (g = −0.388) and RMSSD (g = −0.462). Thus, our failure to detect significant difference in LF/HF was attributable to the small effect size and the inadequate statistical power due to our sample size. Similarly, we found no significant difference in VLF between patients and controls, which could also be due to the reportedly small effect size (g = −0.096 according to Koch et al.^10^).

Similar to activity indices, some of the HRV indices (RRI, HF, and SDNN) reached the statistical significance only in males but not in females. Previous studies have shown sex differences in HRV; males generally show lower HF and greater LF/HF than females in healthy humans, indicating the predominance of sympathetic modulation in males.^11^, ^12^ If depressed patients tend to show lower HF and greater LF/HF, then differences between patients and controls would be smaller in males than in females. In line with this, a study from Germany,^29^ which measured HRV for 30 min in the afternoon while being sedentary in a quiet room, reported that differences in HRV indices between patients with MDD and controls were greater in females than in males, which was the opposite direction to our finding in terms of the sex difference. On the other hand, Kuang et al^12^ reported from China that the effect of depression on HRV was different between males and females in deep breathing and Valsalva test, whereas the effect was not different in the resting and orthostatic test. It was reported in Japanese healthy participants that sex differences in HRV measures varied depending on age and time of the day.^30^ We found negative correlations of age with HRV indices in our controls, which is consistent with previous studies (eg, Umetani et al.^31^). As shown in Figure 1, HRV indices vary substantially by the time of the day. Collectively, the inconsistency between the previous study^29^ and ours may be explained in part by different ethnicities, timing of measurement, and participants' behavior (being sedentary or daily activity). Notably, the sex difference in the effect of depression on HRV might be quantitative, but not qualitative, given that there was no significant interaction between diagnosis and sex in the ANOVA analyses (Table S2). In line, Kuang et al^12^ also failed to find any significant diagnosis x sex interaction in the effect of depression on HRV at rest. To elucidate the sex difference in the relationship between HRV and depression, further studies in different settings will be required.

The simultaneous monitoring of activity and HRV is an advantage of our study. We examined whether the reduced activity correlated with HRV measures. We found no significant correlation for average data of 72 h. However, there were highly significant correlations of L5 activity with RRI (ρ = −0.53, *P* = 0.001) and LF/HF (ρ = 0.44, *P* = 0.007) during L5 in the total participants indicating that dominance of the sympathetic system relative to the parasympathetic system is associated with higher activity during sleep. Importantly, we found significant correlations of LF/HF during L5 with time‐dependent activity measures (L5, M10, and RA) in the patients, suggesting that greater sympathetic load during sleep may have resulted in interrupted sleep, early morning awakening, and reduced daytime activity levels. Since such correlations were not seen in the controls, this might be a characteristic pathology of depression.

There are several limitations in our study. First, the majority of the patients (84%) had taken any psychotropic medications at the time participated in our study. Although we found no significant correlation between activity measures and any medication doses, there was a significant correlation between IMIeq and HRV indices. A number of previous studies have shown the effect of tricyclics on RRI; however, the effects of SSRIs have been reported to be minimal.^6^, ^9^ Since only 2 patients received tricyclics (amitriptyline in a male MDD; nortriptyline in a female MDD), the observed difference in HRV, particularly in males, cannot be attributable simply to the effect of antidepressant medication. Indeed, if the effect of antidepressants played a major role, then we should have obtained significant differences in HRV indices between female patients and female controls as well. Secondly, the number of the participants was not very large, which is subject to type II error. If the number of the participants were increased, statistical differences might have been achieved in the comparisons between female patients and female controls. The possible correlation between activity and HRV indices may become evident with an amplified sample size and more sophisticated statistical analysis such as logistic regression analysis. Thirdly, we included not only patients with MDD, but also those with BD. Since the number of BD patients was small (2 men and 4 women), we could not examine characteristics of BD patients in contrast to MDD patients. Fourthly, the duration of activity monitoring was 3 days in our study that was shorter than the recommended length in previous studies.^21^ It is possible that activity levels between weekends and weekdays differ and thus monitoring for 7 days or more might be required to control for this factor. However, there was no significant difference in duration of data collection in weekends (Saturday and Sunday) between our patients and healthy participants. Finally, we have not performed corrections of p‐values for multiple testing. However, given that there were many significant results in the expected direction, they cannot be ascribed to chance.

In conclusion, we obtained evidence for reduced activity, increased lying/resting time, and reduced HRV indices in male depressed patients, while we failed to obtain significant results in females. The simultaneous monitoring for activity and HRV suggested greater sympathetic load during sleep leads to interrupted sleep and reduced daytime activity levels, which might be a characteristic pathology of depression. These results suggest that combined assessment of HRV and activity by a wearable monitor is useful for the objective assessment of altered physiological functions of major depressive episode. Further studies will be required to draw any conclusion as to the possible sex difference in the effect of depression on activity and HRV indices.

## AUTHOR CONTRIBUTIONS

Kunugi H and Ishimaru designed and supervised the study. Koga N, Kunugi H, and Ishida I recruited participants. Koga N collected data from all participants. Kunugi H and Komatsu Y performed statistical analyses. Shinozaki R provided the wearable monitor and gave technical support. Koga N, Komatsu Y, and Kunugi H wrote the manuscript. All coauthors gave critical comments to the manuscript.

## FUNDING INFORMATION

This work was funded by TOYOBO Co. and NCNP. The funding sources involved only in the financial support.

## CONFLICTS OF INTEREST

Y.K., Y.S. and Y.I. are employees of TOYOBO co. Ltd. S.R. is an employee of Union Tool, co. Ltd.

## APPROVAL OF THE RESEARCH PROTOCOL BY AN INSTITUTIONAL REVIEWER BOARD

The study protocol was approved by the ethics committee at the NCNP (A‐2014151).

## INFORMED CONSENT

Every participant gave written informed consent after receiving the study explanation.

## REGISTRY AND THE REGISTRATION NO. OF THE STUDY/TRIAL

N/A.

## ANIMAL STUDIES

N/A.

## Supporting information


**Data S1** Supplementary methods: Measurement of RRI and 3‐axis acceleration, Analysis of heart rate variability, Analysis of activity magnitude, and Estimation of sleep and awake time

**Table S1** Results of 2‐way (sex and diagnosis) analysis of variance (ANOVA) on activity‐related indices, controlling for age
**Table S2:** Results of 2‐way (sex and diagnosis) ANOVA on activity‐related indices, controlling for age
**Table S3:** Correlations of L5, M10, and RA with HRV indices during L5

## Data Availability

The authors cannot make the data publicly available because of restriction by the ethics committee. However, the data are available, at least in part, upon request.

## References

[1] Germain A , Kupfer DJ . Circadian rhythm disturbances in depression. Hum Psychopharmacol. 2008;23:571–85. 18680211 10.1002/hup.964 PMC2612129

[2] Monteleone P , Martiadis V , Maj M . Circadian rhythms and treatment implications in depression. Prog Neuropsychopharmacol Biol Psychiatry. 2011;2011(35):1569–74. 10.1016/j.pnpbp.2010.07.028 20691746

[3] Tazawaa Y , Wada M , Mitsukura Y , et al. Actigraphy for evaluation of mood disorders: a systematic review and metaanalysis. J Affect Disord. 2019;253:257–69. 31060012 10.1016/j.jad.2019.04.087

[4] Hori H , Koga N , Hidese S , Nagashima A , Kim Y , Higuchi T , et al. 24‐h activity rhythm and sleep in depressed outpatients. J Psychiatr Res. 2016;2(77):27–34. 10.1016/j.jpsychires.2016.02.022 26978182

[5] Malik M . Heart rate variability. Standards of measurement, physiological interpretation, and clinical use. Task Force of the European Society of Cardiology and the North American Society of Pacing and Electrophysiology. Eur Heart J. 1996;17:354–81. 8737210

[6] Alvares GA , Quintana DS , Hickie IB , Guastella AJ . Autonomic nervous system dysfunction in psychiatric disorders and the impact of psychotropic medications: a systematic review and meta‐analysis. J Psychiatry Neurosci. 2016;41:89–104. 26447819 10.1503/jpn.140217 PMC4764485

[7] Choi KW , Jeon HJ . Heart rate variability for the prediction of treatment response in major depressive disorder. Front Psych. 2020;11:607. 10.3389/fpsyt.2020.00607 PMC7339656 32695031

[8] Faurholt‐Jepsen M , Kessing LV , Munkholm K . Heart rate variability in bipolar disorder: A systematic review and meta‐analysis. Neurosci Biobehav Rev. 2017;73:68–80. 27986468 10.1016/j.neubiorev.2016.12.007

[9] Kemp AH , Quintana DS , Gray MA , Felmingham KL , Brown K , Gatt JM . Impact of depression and antidepressant treatment on heart rate variability: a review and meta‐analysis. Biol Psychiatry. 2010;67(11):1067–74. 20138254 10.1016/j.biopsych.2009.12.012

[10] Koch C , Wilhelm M , Salzmann S , Rief W , Euteneuer F . A meta‐analysis of heart rate variability in major depression. Psychol Med. 2019 Sep;49(12):1948–57. 31239003 10.1017/S0033291719001351

[11] Koenig J , Thayer JF . Sex differences in healthy human heart rate variability: a meta‐analysis. Neurosci Biobehav Rev. 2016;64:288–310. 26964804 10.1016/j.neubiorev.2016.03.007

[12] Kuang D , Cui L , Kuang S , Yang R , Chen X , Zhang L , et al. Effect of gender‐related depression on heart rate variability during an autonomic nervous test. Psychiatry Res. 2019;272:258–64. 30594758 10.1016/j.psychres.2018.12.099

[13] Otsubo T , Tanaka K , Koda R , et al. Reliability and validity of Japanese version of the Mini‐International Neuropsychiatric Interview. Psychiatry Clin Neurosci. 2005;59:517–26. 16194252 10.1111/j.1440-1819.2005.01408.x

[14] Sheehan DV , Lecrubier Y , Sheehan KH , et al. The Mini‐International Neuropsychiatric Interview (M.I.N.I.): the development and validation of a structured diagnostic psychiatric interview for DSM‐IV and ICD‐10. J Clin Psychiatry. 1998;59(Suppl 20):22–33. quiz 34‐57. 9881538

[15] American Psychiatric Association . Diagnostic and Statistical Manual of Mental Disorders. 5th ed. Arlington: American Psychiatric Publishing; 2013. p. 2013.

[16] Hamilton M . Development of a rating scale for primary depressive illness. Br J Soc Clin Psychol. 1967;6:278–96. 6080235 10.1111/j.2044-8260.1967.tb00530.x

[17] Williams JB , Kobak KA , Bech P , et al. The GRID‐HAMD: standardization of the Hamilton Depression Rating Scale. Int Clin Psychopharmacol. 2008;23(3):120–9. 18408526 10.1097/YIC.0b013e3282f948f5

[18] World Medical Association . World Medical Association Declaration of Helsinki: ethical principles for medical research involving human participants. JAMA. 2013;310(20):2191–4. 24141714 10.1001/jama.2013.281053

[19] Young RC , Biggs JT , Ziegler VE , Meyer DA . A rating scale for mania: reliability, validity and sensitivity. Br J Psychiatry. 1978 Nov;133:429–35. 728692 10.1192/bjp.133.5.429

[20] Inada T , Inagaki A . Psychotropic dose equivalence in Japan. Psychiatry Clin Neurosci. 2015;69(8):440–7. 25601291 10.1111/pcn.12275

[21] Luik AI , Zuurbier LA , Direk N , Hofman A , Van Someren EJ , Tiemeier H . 24‐hour activity rhythm and sleep disturbances in depression and anxiety: a population‐based study of middle‐aged and older persons. Depress Anxiety. 2015;32(9):684–92. 25693731 10.1002/da.22355

[22] Goncalves BS , Adamowicz T , Louzada FM , Moreno CR , Araujo JF . A fresh look at the use of nonparametric analysis in actimetry. Sleep Med Rev. 2015;20:84–91. 25065908 10.1016/j.smrv.2014.06.002

[23] Kochiya Y , Hirabayashi A , Ichimaru Y . Nocturnal heart rate variability in 1‐year‐old infants analyzed by using the Least Square Cosine Spectrum Method. J Physiol Anthrop. 2017;36:36. 10.1186/s40101-017-0152-8 PMC5603050 28915915

[24] Hirabayashi A , Kochiya Y , Ichimaru Y . Examination and application of estimation method for Body postures in daily life using triaxial accelerometers. Jap J Physiolol Anthropol. 2015;20:187–96. (in Japanese).

[25] Kidwell M , Ellenbroek BA . Heart and soul: heart rate variability and major depression. Behav Pharmacol. 2018;29:152–64. 29543649 10.1097/FBP.0000000000000387

[26] Shaffer F , McCraty R , Zerr CL . A healthy heart is not a metronome: an integrative review of the heart's anatomy and heart rate variability. Front Psychol. 2014 Sep;30(5):1040. 10.3389/fpsyg.2014.01040 PMC4179748 25324790

[27] Ohayon MM , Carskadon MA , Guilleminault C , Vitiello MV . Meta‐analysis of quantitative sleep parameters from childhood to old age in healthy individuals: developing normative sleep values across the human lifespan. Sleep. 2004;27(7):1255–73. 15586779 10.1093/sleep/27.7.1255

[28] Armitage R , Hoffmann R , Emslie G , Rintelman J , Moore J , Lewis K . Rest‐activity cycles in childhood and adolescent depression. J Am Acad Child Adolesc Psychiatry. 2004;43:761–9. 15167093 10.1097/01.chi.0000122731.72597.4e

[29] Voss A , Boettger MK , Schulz S , Gross K , Bär KJ . Gender‐dependent impact of major depression on autonomic cardiovascular modulation. Prog Neuropsychopharmacol Biol Psychiatry. 2011;35:1131–8. 21453741 10.1016/j.pnpbp.2011.03.015

[30] Yamasaki Y , Kodama M , Matsuhisa M , Kishimoto M , Ozaki H , Tani A , et al. Diurnal heart rate variability in healthy subjects: effects of aging and sex difference. Am J Physiol. 1996;271:H303–10. 8760189 10.1152/ajpheart.1996.271.1.H303

[31] Umetani K , Singer DH , McCraty R , Atkinson M . Twenty‐four hour time domain heart rate variability and heart rate: relations to age and gender over nine decades. J Am Coll Cardiol. 1998;31:593–601. 9502641 10.1016/s0735-1097(97)00554-8

