# Supplementary material for: Simultaneous monitoring of activity and heart rate variability in depressed patients: A pilot study using a wearable monitor for 3 consecutive days
Source: Neuropsychopharmacol Rep. 2022 Jul 29;42(4):457–67. doi: 10.1002/npr2.12285 (PMC9773773; doi:10.1002/npr2.12285)
Supplement: Supplementary file 1 — Data S1 Supplementary methods: Measurement of RRI and 3‐axis acceleration, Analysis of heart rate variability, Analysis of activity magnitude, and Estimation of sleep and awake time [file NPR2-42-457-s001.docx]

**Supplementary methods**

**Measurement of RRI and 3-axis acceleration**

The RR intervals (RRI) and 3-axis acceleration were simultaneously measured using the wearable heart rate sensor (WHS-1, Union Tool Co., Tokyo, Japan). Measurement was conducted under free-living conditions from 3 p.m. for 3 consecutive days (72 hours). The participants wore the device on their left chest with disposable electrodes (BlueSensor, Ambu, Copenhagen, Denmark). The device has two springs of snap to connect disposable electrodes which induct two body surface potentials to WHS-1. Electrocardiogram obtained through differential of the body surface potentials are processed by the analog band path filter of WHS-1, to detect the peak position of R wave. The processed electrocardiogram is sampled by A/D converter with 10 bits and 1 kHz. Therefore, the RRI is measured by using the peak position of R wave with resolution of 1 ms. The 3-axis acceleration is measured by MEMS (micro electro mechanical systems) accelerometer with 32 Hz and 8 bits in a dynamic range of ± 4 G, where the unit “G” is the gravitational acceleration. WHS-1 records a maximum acceleration every 4 s.

**Analysis of heart rate variability**

The RRI and 3-axis acceleration data stored in WHS-1 were transferred to a computer to analyze the data. The software of “RRI Analyzer” (Union Tool Co., Tokyo, Japan) was used. In the software, two noise filters are applied to eliminate artifact of the RRI time series. With the first filter, the RRI of above 2000 ms and less than 300 ms was eliminated as the artifact. With the second filter, the RRI different from average heart rate (HR) was eliminated in the following manner. Average HR, which is the instantaneous HR calculated from RRI, is an 8-HR moving average without over 15 bpm from one HR before. Subsequently, RRI above 30 bpm from the average HR was eliminated.

Also, the artifact eliminated RRI was analyzed with frequency domain in the following procedure. The artifact eliminated RRI was interpolated with spline function, then the interpolated RRI was resampled with the interval of 100ms. To remove direct current and extremely low frequency from the interpolated RRI, quadratic function g(ti)=ati^2+bti+c was extracted from the time series of resampled RRI as f(ti) by using least-square approximation. Here, i is index which increased every 100ms, and a, b and c are constant. A function h(ti) which removed direct current and extremely low frequency from the interpolated RRI was obtained with h(ti)=f(ti)-g(ti). Subsequently, power spectral densities (PSD) were calculated from function h(ti) multiplied by the Hanning window with fast Fourier transform (FFT) for 180s every 10s, and quantified by measuring the area in two frequency bands: 0.04 to 0.15 Hz (low frequency, LF), and 0.15 to 0.4 Hz (high frequency, HF). The HF reflects the parasympathetic activity, and the LF/HF ratio reflects the sympathovagal balance. From RRI without the artifact in the time-domain, coefficient of variation of RRI (CVRR) was calculated. In this study, HF > 5000 and LF/HF > 20 were considered outliers and excluded from the analysis. Also, RRI and CVRR, which were in the time zone of the outlier HF and LF/HF, were excluded. After excluding the all outlier, average RRI and HF, LF/HF, CVRR were calculated. We also obtained the average RRI, HF, LF/HF, and CVRR every 30 min.

**Analysis of activity magnitude**

WHS-1 has a 3-axis acceleration sensor. The X-axis acceleration is represented for left-right acceleration, Y-axis for superior-inferior, and Z-axis for anterior-posterior. The square root of sum of squares of the 3-axis acceleration was used as activity magnitude. The formula of it is as follows:

Activity magnitude = $\sqrt{\mathbf{x}^{\mathbf{2}}\mathbf{+}\mathbf{y}^{\mathbf{2}}\boldsymbol{+}\mathbf{z}^{\mathbf{2}}}$ $\boldsymbol{-1}$ (1)

The average for every 30 min were also calculated to verify the 24-hour change in activity magnitude.

**Estimation of sleep and awake time**

Hirabayashi et al (2015) and Kochiya et al (2017) have reported that the participants were in a supine position if the Y-axis acceleration was greater than -0.7G. We also confirmed that. However, the participants in a supine position may be sleeping, or may only be lying down and resting. Therefore, it was hypothesized that the supine posture would continue for more than 1 hour if sleeping, and the time during which the Y-axis acceleration was greater than -0.7G for 1 hour or more was determined. The method of calculating the time when the Y-axis acceleration is greater than -0.7G is as follows. We determined the participant is lying time, when data of Y-axis acceleration was equal to or greater then -0.7G for 10 minutes or longer. Morphology operation was applied after binarization processing for Y axis acceleration, for shortening analysis time. The details of morphology operation are as follows. First, Y-axis acceleration was replaced by the value of 1 to less than -0.7G, the value 0 to 0.7G or over in time, for time series data of Y-axis acceleration, to binarize. Then, morphology operation was applied, which conducted opening processing and closing processing for 120s and 300s, to binarized Y-axis acceleration time series data. Analysis time could be shortened by combining an expansion operation and a contraction operation in this manner, and makes it easier to extract the outline of Y-axis acceleration time series data, excluding the posture changing in a short time. In these ways, we determined lying time over 1 hour. Then, of the calculated time, the longest time in 24 hours from 18:00 was estimated as the sleeping time. If the time interval between the supine posture lasting more than 1 hour and the time interval between the next supine posture lasting more than 1 hour was less than 1 hour, the time interval was considered to be the time interval during which the participants had stopped sleeping in the toilet or the like, and all periods around the time were estimated to be the sleeping time. Sleep time estimated from Y-axis acceleration was correlated with self-reported sleep time. （r=.608, p<0.01）Time other than the sleep time was considered as the awake time.

References

Hirabayashi A, Kochiya Y, Ichimaru Y. Examination and application of estimation method for Body postures in daily life using triaxial accelerometers. *Jap J Physiolol Anthropol.* 2015; 20: 187.

Kochiya Y, Hirabayashi A, Ichimaru Y. Nocturnal heart rate variability in 1-year-old infants analyzed by using the Least Square Cosine Spectrum Method. *J Physiol Anthrop*. 2017; 36: 36
