# Supplementary material for: Simultaneous monitoring of activity and heart rate variability in depressed patients: A pilot study using a wearable monitor for 3 consecutive days
Source: Neuropsychopharmacol Rep. 2022 Jul 29;42(4):457–67. doi: 10.1002/npr2.12285 (PMC9773773; doi:10.1002/npr2.12285)
Supplement: Supplementary file 2 — Table S1 Results of 2‐way (sex and diagnosis) analysis of variance (ANOVA) on activity‐related indices, controlling for age Table S2: Results of 2‐way (sex and diagnosis) ANOVA on activity‐related indices, controlling for age Table S3: Correlations of L5, M10, and RA with HRV indices during L5 [file NPR2-42-457-s002.docx]

**Supplementary Table S1**

Results of 2-way (sex and diagnosis) analysis of variance (ANOVA) on activity-related indices, controlling for age

|  | Patients | Controls | Effect | | | |
| --- | --- | --- | --- | --- | --- | --- |
|  | （7M, 12F) | （9M, 9F) | Age | Sex | Diagnosis | Sex X diagnosis |
|  | Mean （SD） | Mean （SD） | p-value (η^2^) | p-value (η^2^) | p-value (η^2^) | p-value  (η^2^) |
| 1. ACT | | | | | | |
| Total | 0.140  (0.027) | 0.167  (0.038) | p=0.66  (0.01) | p=0.41  (0.02) | **p=0.018**  (0.16) | p=0.31  (0.03) |
| Male | 0.138  (0.011) | 0.177  (0.047) |  |  |  |  |
| Female | 0.140  (0.033) | 0.158  (0.024) |  |  |  |  |
| 2.TRT (min) | | | | | | |
| Total | 545.0  (138.6) | 483.3  (117.4) | p=0.23  (0.04) | p=0.97  (0.00) | p=0.18  (0.05) | P=0.66  (0.01) |
| Male | 537.1  (110.0) | 500.4  (130.1) |  |  |  |  |
| Female | 550.3  (159.4) | 467.9  (109.2) |  |  |  |  |
| 3. L5 | | | | | | |
| Total | 0.040  (0.021) | 0.036  (0.015) | p=0.42  (0.02) | p=0.22  (0.05) | p=0.35  (0.03) | p=0.34  (0.03) |
| Male | 0.048  (0.007) | 0.036  (0.017) |  |  |  |  |
| Female | 0.035  (0.025) | 0.036  (0.013) |  |  |  |  |
| 4. M10 | | | | | | |
| Total | 0.206  (0.045) | 0.253  (0.077) | p=0.91  (0.00) | p=0.31  (0.03) | **p=0.034**  (0.13) | p=0.27  (0.04) |
| Male | 0.205  (0.020) | 0.276  (0.099) |  |  |  |  |
| Female | 0.207  (0.055) | 0.229  (0.040) |  |  |  |  |
| 5. RA | | | | | | |
| Total | 0.68  (0.15) | 0.73 (0.12) | p=0.66  (0.01) | p=0.40  (0.02) | p=0.12  (0.07) | p=0.26  (0.04) |
| Male | 0.62 (0.06) | 0.75 (0.15) |  |  |  |  |
| Female | 0.71  (0.17) | 0.72  (0.09) |  |  |  |  |

Continuous variables that were not significantly deviated from the normal distribution were subject to the ANOVA analysis. A significant p-value is shown in bold cases.

ACT: mean activity per day; TRT: mean total resting time per day

L5: average activity across the least active 5-hour period

M10: average activity during the most active 10-hour period

RA: relative amplitude of the rest-activity rhythm

**Supplementary Table S2**

**Results of 2-way (sex and diagnosis) ANOVA on activity-related indices, controlling for age**

|  | Patients | Controls | Effect | | | |
| --- | --- | --- | --- | --- | --- | --- |
|  | （7M, 12F) | （9M, 9F) | Age | Sex | Diagnosis | Sex X diagnosis |
|  | Mean （SD） | Mean （SD） | p-value (η^2^) | p-value (η^2^) | p-value (η^2^) | p-value  (η^2^) |
| 1. RRI | | | | | | |
| Total | 782.0  (108.1) | 883.4  (97.8) | p=0.50  (0.01) | **p=0.003**  (0.24) | **p=0.001**  (0.32) | p=0.36  (0.03) |
| Male | 700.1  (79.6) | 851.1  (78.9) |  |  |  |  |
| Female | 829.7  (94.5) | 915.8  (108.3) |  |  |  |  |
| 2. HF | | | | | | |
| Total | 508.5  (536.5) | 675.3  (373.5) | **p=0.028**  (0.14) | **p=0.073**  (0.10) | p=0.12  (0.08) | p=0.55  (0.01) |
| Male | 299.4  (236.7) | 652.8  (266.3) |  |  |  |  |
| Female | 630.4  (629.6) | 697.9  (473.7) |  |  |  |  |
| 3. SDNN | | | | | | |
| Total | 39.1  (18.0) | 50.0  (12.1) | **p=0.006**  (0.21) | p=0.40  (0.02) | **p=0.012**  (0.18) | p=0.63  (0.01) |
| Male | 36.0  (16.9) | 52.1  (11.6) |  |  |  |  |
| Female | 40.9  (19.0) | 47.9  (12.9) |  |  |  |  |
| 4. RMSSD | | | | | | |
| Total | 30.2  (17.9) | 37.7  (15.0) | **p=0.037**  (0.;13) | **p=0.043**  (0.12) | p=0.072  (0.10) | p=0.93  (0.00) |
| Male | 23.5  (12.7) | 34.7  (9.8) |  |  |  |  |
| Female | 34.1  (19.8) | 40.7  (19.0) |  |  |  |  |
| 5. CVRR | | | | | | |
| Total | 5.24  (2.08) | 6.21  (1.24) | **p=0.003**  (0.24) | p=0.82  (0.00) | **p=0.040**  (0.13) | p=0.73  (0.00) |
| Male | 51.6  (2.13) | 6.60  (1.10) |  |  |  |  |
| Female | 5.28  (2.14) | 5.82  (1.31) |  |  |  |  |
| 6. NN50 | | | | | | |
| Total | 6.79  (7.25) | 8.03  (5.00) | **p=0.003**  (0.24) | p=0.11  (0.08) | p=0.29  (0.04) | p=0.98  (0.00) |
| Male | 5.11  (4.45) | 7.72  (3.44) |  |  |  |  |
| Female | 7.77  (8.50) | 8.33  (6.41) |  |  |  |  |
| 7. pNN50 | | | | | | |
| Total | 11.21  (12.33) | 13.94  (9.19) | **p=0.005**  (0.23) | p=0.055  (0.11) | p=0.20  (0.05) | p=0.87  (0.00) |
| Male | 7.29  (6.16) | 13.00  (6.56) |  |  |  |  |
| Female | 13.50  (14.58) | 14.89  (11.60) |  |  |  |  |

Continuous variables that were not significantly deviated from the normal distribution were subject to the ANOVA analysis. A significant p-values are shown in bold cases.

**Supplementary Table S3**

Correlations of L5, M10, and RA with HRV indices during L5

| Activity | | RRI | HF | LFHF | CVRR | SDNN | RMSSD | NN50 | pNN50 |
| --- | --- | --- | --- | --- | --- | --- | --- | --- | --- |
| L5 | ρ | -.536 | -.241 | .435 | -.077 | -.293 | -.287 | -.276 | -.314 |
|  | P | **0.001** | .151 | **0.007** | 0.65 | 0.078 | 0.086 | 0.099 | 0.058 |
| M10 | ρ | .295 | .234 | -.137 | .204 | .195 | .155 | .091 | .104 |
|  | P | 0.076 | 0.16 | 0.42 | 0.23 | 0.25 | 0.36 | 0.59 | 0.54 |
| RA | ρ | .576 | .318 | -.478 | .159 | .330 | .303 | .266 | .305 |
|  | P | **0.000** | 0.055 | **0.003** | 0.35 | **0.046** | 0.068 | 0.11 | 0.066 |

Correlations by Spearman. Significant p-values are shown in bold cases.

Correlation with VLF and ULF are omitted.
